# Factors Affecting Utilization of Healthcare Services Among Nasahablood and Ayah 4 Internally Displaced Persons in Hargeisa, Somaliland

**DOI:** 10.1002/puh2.70140

**Published:** 2025-10-09

**Authors:** Ayan Hussein Korse, Vitalis Okoth Odero, Mohamed Said Hassan, Hana Mahdi Dahir, Khadar Abdi Ibrahim

**Affiliations:** ^1^ School of Postgraduate Studies and Research Amoud University Borama Somalia; ^2^ College of Health Sciences School of Medicine and Surgery Amoud University Borama Somalia

**Keywords:** health equity, internally displaced persons (IDPs), utilization

## Abstract

**Background:**

Globally, 4.5 billion people lack full coverage of essential health services, and displaced populations are disproportionately affected. Understanding the specific barriers they face is critical for designing effective health interventions.

**Objective:**

To identify the factors affecting the utilization of healthcare services among internally displaced persons (IDPs) in Hargeisa, Somaliland.

**Design:**

A community‐based, cross‐sectional study.

**Site:**

The Nasahablood and Ayah 4 IDP camps in Hargeisa, Somaliland.

**Participants:**

A proportionate stratified sample of 271 households.

**Main Measures:**

The primary outcome was the level of healthcare utilization (low, moderate, and high), analyzed using ordinal logistic regression (OLR). Predictor variables included accessibility, affordability, and socioeconomic factors.

**Results:**

A majority of respondents (73.5%) reported low healthcare utilization, with only 14.4% reporting high utilization. OLR revealed that affordability was the most significant barrier to care (*p* < 0.001), followed by physical accessibility (*p* = 0.025). Both factors were associated with substantially lower odds of health service utilization. Additionally, after controlling for other variables, gender emerged as a significant predictor, with male respondents having lower odds of utilizing healthcare services compared to female respondents (*p* = 0.030).

**Conclusion:**

Healthcare underutilization in these IDP camps is primarily driven by powerful structural barriers of cost and distance, compounded by gender‐specific factors. We recommend targeted interventions, including fee‐exemption policies and mobile health clinics, to address these critical gaps and improve health outcomes for this vulnerable population.

## Graphical abstract

GRAPHICAL ABSTRACT

A community‐based study of 271 households in the Nasahablood and Ayah 4 IDP camps in Hargeisa, Somaliland, revealed critically low healthcare utilization, with 73.5% of respondents reporting low use. Ordinal logistic regression identified three significant structural barriers: lack of **affordability** (*p* < 0.001) as the primary obstacle, followed by poor physical **accessibility** (*p* = 0.025), and **gender**‐specific factors, with women facing lower odds of utilization (*p* = 0.030). These findings highlight the urgent need for targeted interventions, such as fee‐exemption policies and mobile clinics, to improve health equity for this vulnerable population.

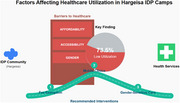

## 1 Introduction

According to the World Health Organization (WHO) in 2021, approximately 4.5 billion people were not fully covered by essential health services [1]. Approximately 2 billion people face financial hardship, including one billion experiencing catastrophic out‐of‐pocket health spending globally. By the conclusion of 2022, a staggering 108.4 million individuals across the globe were displaced against their will due to factors such as persecution, conflict, violence, human rights violations, and disruptive events that severely impact public order. This figure reflects an alarming rise of 19 million people compared with the end of 2021, exceeding the entire population of countries such as Ecuador, the Kingdom of the Netherlands, and Somalia. Furthermore, this increase represents the most significant year‐on‐year surge in forced displacement recorded in statistics provided by the United Nations High Commissioner for Refugees [2]. The Norwegian Refugee Council stated that there were 26.4 million internally displaced people at the end of 2011, including 3.5 million displaced people last year. The WHO aims to ensure that everyone can access the health services they need without financial hardship.

In Africa, the accessibility and coverage of essential health services are especially low. Only 43% of pregnant women attended the four recommended prenatal visits, compared to the global average of 55% in 2014. Only 49% of births are attended by skilled health personnel, compared to the global average of 70% [3]. Some families were displaced nearly 30 years ago, whereas others continued to arrive in the city daily because of conflicts and natural disasters. Families that have moved to these areas live in precarious conditions and are unable to meet basic needs due to inconsistent health service provision or exclusion from accessing humanitarian support due to conflicts in the city [4]. The study identified multiple barriers to maternal and child health service utilization in internally displaced person (IDP) settings, categorized into individual, interpersonal, community, organizational, and policy levels, including economic constraints, cultural beliefs, lack of privacy in facilities, and poor healthcare infrastructure [5, 6].

This model posits that healthcare utilization is determined by the interplay between the health system and the patient across several key dimensions. Central to this investigation are affordability, which reflects an individual's financial capacity to pay for services, and accessibility, which considers the geographic ease of reaching care. An individual's socioeconomic status directly impacts these dimensions as high costs or long distances can create significant barriers that hinder the use of health services. This framework therefore provides a robust structure for examining how these factors collectively influence healthcare utilization among the IDP population in Hargeisa.

Internally displaced people in Hargeisa, Somaliland, face a complex set of challenges that directly contribute to poor child health outcomes like stunting. The study highlights significant barriers to healthcare, evidenced by low rates of full vaccination, inadequate deworming practices, and a high prevalence of home deliveries instead of deliveries at health facilities. These issues are compounded by critical socioeconomic factors, including widespread maternal illiteracy (86.4%) and poor maternal nutrition, such as insufficient extra food intake during lactation [7]. The health coverage for Somalia's approximately three million IDPs is severely compromised in a country with a national ratio of just 0.023 doctors per 1000 people [8]. IDPs in Somalia face significant challenges utilizing maternal and child health services due to barriers like armed conflict, low income, and lack of female autonomy, in a nation with a maternal mortality ratio of 692 deaths per 100,000 live births. Based on the provided reports, Somalia's IDPs face severe barriers to health service utilization, with poverty being a major obstacle as 55.3% of the IDP population in Somaliland lives below the poverty line [9]. Healthcare utilization for IDPs in Somaliland is severely hampered by economic hardship, with an estimated 55.3% of the IDP population living below the poverty line [10].

A specific contextual factor that distinguishes the IDP populations in Ayah 4 and Nasahablood is the documented, severe underutilization of their designated primary health centers, indicating acute barriers beyond simple physical access. Although other regions may lack facilities, these camps have them but are not using them, as evidenced by a significant 6‐month decline in OPD visits. The Ayah 4 Health Center experienced drops as high as 41.6% in April, whereas the Nasahablood Health Center saw a staggering 47.83% decline in June alone, suggesting unique and pressing local challenges, such as prohibitive costs, lack of trust in service quality, or the prioritization of more immediate survival needs like food and water.

Ayah 4 Health Center is located in Ahmed Dhagah district in Hargeisa, Somaliland, and serves approximately 400 households, whereas Nasahablood is located in G. Libah District and serves 550 households. Health centers are considered the primary points of care for local communities. It offers various departments, including an outpatient department, EPI services, a pharmacy, ANC, and a delivery center, providing essential healthcare services to the population it serves [11]. OPD visits decreased by 16.2%, 33.1%, 7.2%, 41.6%, 17.8%, and 8.1% in January, February, March, April, May, and June, respectively. This indicates that declining utilization among people living in the Ayah 4 IDP Camp may not fully utilize the healthcare services offered by the Ayah 4 Health Center despite it being the primary point of care for the local community. Although the Nasahablood Health Center OPD visits dropped by 31.41%, 8.41%, 6.12%, 13.04%, 13.75%, and 47.83% in January, February, March, April, May, and June, respectively. This information indicates a decline in the utilization of healthcare services at the Nasahablood C Health Center among people living in the Nasahablood C IDP camp.

## 2 Methods

### 2.1 Study Design

This study adopted a cross‐sectional survey design and was conducted in IDP camps. Data were collected from the households using structured questionnaires that determine the factors that affect the utilization of healthcare services. A cross‐sectional study design is a technique for collecting data from a target population at one point. It has the advantage of saving time and enabling all patients to be easily targeted. In a cross‐sectional survey, data on multiple variables were collected from a large sample at a specific point in time. This ensured that enough data were collected relatively quickly. A cross‐sectional survey design enabled the researcher to assess the factors affecting the utilization of healthcare services among Nasahablood and Ayah 4 IDPs in Hargeisa and Somaliland.

### 2.2 Target Population

The study's target population was Nasahablood and Ayah 4 IDPs, Ayah 4 IDP, 400 households, and Nasahablood C, 550 households. This population was chosen because the utilization of healthcare services was a primary concern for the Nasahablood and Ayah 4 IDP camps [12].

### 2.3 The Rationale for Selecting the Two Camps

The selection of the Nasahablood and Ayah 4 IDP camps was purposive, guided by preliminary data from the Ministry of Health Development (MoHD) that revealed a significant and documented decline in healthcare utilization, marking these as priority sites for investigation. This choice was further strategic, as the two camps represent different settlement contexts: Nasahablood is a long‐established camp, whereas Ayah 4 has been more recently expanded. This dual‐site approach allows for a comparative analysis of potential barriers to healthcare, offering insights into whether challenges differ based on the maturity and stability of the IDP settlement.

### 2.4 Sample Size

The sample size for this study was 271 households, calculated using the methodology [13]. This calculation was based on a 95% confidence interval, a 5% margin of error, and a conservative 50% prevalence rate, the latter chosen due to the lack of previous similar studies in the Hargeisa region. To ensure the sample was representative of the target populations, this total of 271 households was then allocated proportionally between the two camps, resulting in surveying 157 households from the larger Nasahablood camp (total 550 households) and 114 households from the Ayah 4 camp (total 400 households).

Z = confident interval 95%,

*p* = Based on 50% prevalence rate *n* = 271

Sample size $n=\mathrm{DEFF}\times \;[\;Np\left(1-p\right)]\;/\;[\;\left(d^{2}/Z^{2}\right)\times \left(N-1\right)+p\left(1-p\right)]$

### 2.5 Sampling Technique

The study utilized a stratified sampling technique to select a sample of households. As described, this technique involves dividing the population into subgroups or strata and selecting a sample from each. The strata were the Nasahablood C and Ayah 4 IDPs, and a proportionate sample of participants was chosen. Within each stratum, simple random sampling was used to select a specific household for inclusion.

### 2.6 Data Analysis

Ordinal logistic regression (OLR) analysis was used as a data analysis technique. Data were coded, cleaned, edited, and entered into SPSS data to minimize logical errors and design skipping patterns. Data were exported to the STATA window for analysis. Descriptive analysis and OLR were performed by computing the proportions and summary statistics. The information is then presented using simple frequencies, summary measures, tables, and figures. Inferential statistical regression analysis was employed to evaluate the effect of the independent variables (IVs) and dependent variables (DV). Regression analysis is commonly utilized when a study focuses on predicting a variable based on other factors. Given that the DV is in ordinal form, an OLR was used to analyze the effect between the independent and DV.

### 2.7 Study Variables and Their Measurement

#### 2.7.1 Outcome Variable

The study outcome variable was the utilization of healthcare services. The IDP center has health centers that provide various primary healthcare services, including antenatal care, laboratory tests, and gynecological tests. This indicates a moderate utilization level, as the center caters to basic health needs but may not have the full spectrum of specialized services. The IDP center has various health facilities, including an MCH/health center, outpatient clinics, and specialized clinics/hospitals. This finding suggests a high level of utilization. The health center in the IDP center has a health center with available services, indicating a low utilization of healthcare services.

#### 2.7.2 Independent Variable

The IVs for this study were socioeconomic status, accessibility, and affordability, and socioeconomic factors were education level, gender, and household income. The inaccessibility factors were measured geographical distance and type of transportation. The affordability factor was measured the cost of healthcare.

## 3 Results

### 3.1 Utilization of Healthcare Services

**FIGURE 1:**
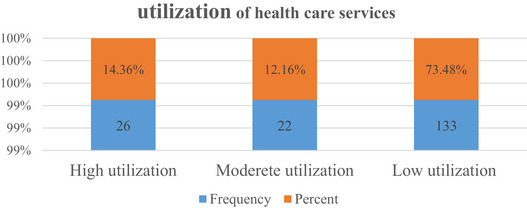
Utilization of healthcare services.

The chart depicts the frequency and percentage distribution of healthcare service utilization levels within a sample population. The majority (73.48%) demonstrate low utilization, with a frequency of 133. Moderate utilization is observed in 12.16% of the cohort, represented by a frequency of 22, whereas high utilization comprises 14.36%, with a frequency of 26. This distribution suggests that the predominant pattern among individuals in the sample population is low‐level utilization of healthcare services (Figure 1).

#### 3.1.1 Descriptive Statistics

The outcome of descriptive analysis: The age of representative shows the distribution of respondents across various age groups. The data show that the highest proportion of respondents dropped within the 30–40 years age group, representing *n* = 112 (41.3%) of the entire respondents, whereas the 20–30 years age group, comprising 64 (23.6%) age groups of 18–19 years and above 50 years, accounted for *n* = 15 (5.5%) and *n* = 80 (29.5%)—sources of income for the respondent. Most respondents (*n* = 216, 79.70%) depended on formal employment as a source of income. In comparison, financial support from family members/friends was utilized by 44 (16.20%) of the representatives, highlighting the role of personal connections in providing economic assistance. A small percentage (*n* = 1, 0.40%) relied on social support networks for financial aid. Humanitarian assistance was used by 10 (3.70%) respondents, indicating a moderate level of reliance on external aid for financial support—the marital status. The data show that most respondents were married, accounting for 194 (71.3%) of the surveyed population. By contrast, single individuals constituted 41 (15.1%) respondents, highlighting a notable portion of unmarried individuals. Although 28 (10.3%) respondents were divorced, this indicates a small but still significant proportion of individuals who had an adept marital dissolution. Separate individuals accounted for the smallest percentage at *n* = 9 (3.3%). The data show that most representatives were from Nasahablood IDP camps (*n* = 148, 54.60%). This indicates a higher sample rate in the Nasahablood IDP camp, whereas the Ayah 4 IDP camp representatives were 123 (45.40%). The data also indicated that a small percentage of respondents (*n* = 15, 5.54%) received health support from UNHCR or INGO, whereas the majority (*n* = 256, 94.46%) did not receive such support (Table 1).

**TABLE 1:** Descriptive statistics.

Variable/Background information	Categories	Frequency	Percentage
Age of respondent	18–19 years	15	5.6
	20–30 years	64	23.6
	30–40 years	112	41.3
	Above 50 years	80	29.5
Sources income	Social support network	1	0.40
	Formal employment	216	79.70
	Financial support from family members/friends	44	16.20
	Humanitarian assistances	10	3.70
Marital status	Single	41	15.1
	Married	194	71.3
	Divorce	28	10.3
	Separated	9	3.3
The residence	Ayah 4 IDP camp	123	45.40
	Nasahablood C IDP camp	148	54.60
Support from UNHCR/INGO	Yes No	15 256	5.54 94.46
Accessibility Geographic distance	0–1000 km	215	79.33
	0–2 km	43	15.87
	More than 3 km	13	4.80
Type of transportation	Waking	177	97.79
	Taxi	1	0.55
	Public transport	3	1.66
Gender	Male	55	20.30
	Female	216	79.70
Education level	Informal education	59	21.77
	Illiteracy	134	49.45
	Primary/Intermediate	45	16.6
	Secondary	24	8.86
	Bachelor	9	3.32
Household income per month	Less than $50	17	6.27
	$50–$100	212	78.23
	$200	34	12.55
	$300	5	1.85
Affordability healthcare cost	Registration fee	61	33.70
	Laboratory investigations	4	2.21
	Medication	24	13.26
	All of them	91	50.28
	Registration and lab	1	0.55

Abbreviation: IDP, internally displaced person.

#### 3.1.2 Bivariate Analysis OLR

Accessibility and affordability are important determinants in the utilization of healthcare services, with inaccessibility having a significant negative coefficient (Coef. = −1.441912, *p* = 0.025), showing that the lack of access significantly slows healthcare services. In contrast, the unaffordable category has the most significant negative coefficient (Coef. = −2.270762, *p* = 0.000), focusing on affordability as a substantial obstacle in healthcare services. Overall, these results indicate that a combination of gender, accessibility, and affordability affects the utilization of healthcare services (Table 2).

**TABLE 2:** Bivariate analysis ordinal logistic regression.

Variable	Utilization rata			*p* value	Coef.
**Respondent age**	High no. (%)	Low no. (%)	Moderate no. (%)		
**Respondent age**					
18–19 years	1 (8.33%)	8 (66.67%)	3 (25.00%)		
20–30 years	3 (7.50%)	34 (85.00%)	3 (7.50%)	0.221	0.9023257
30–40 years	17 (21.52%)	54 (68.35%)	8 (10.13%)	0.850	−0.1203686
Above 50 years	5 (10.00%)	37 (74.00%)	8 (16.00%)	0.694	0.2637258
**Respondent residence**					
Nasahablood C IDP	10 (9.71%)	79 (76.70%)	14 (13.59%)	0.167	0.4630163
Ayah 4 IDP	16 (20.51%)	54 (69.23%)	8 (10.26%)		
**Sources income**					
Formal employment	11 (8.09%)	108 (79.41%)	17 (12.50%)	2.61	1.775624
Financial support from family members/friends	11 (32.35%)	20 (58.82%)	3 (8.82%)	0.402	0.6127359
Humanitarian assistances	4 (44.44%)	4 (44.44%)	1 (11.11%)		
**Support from UNHCR/INGO**					
Yes	3 (33.33%)	3 (33.33%)	3 (33.33%)		
No	23 (13.37%)	130 (75.58%)	19 (11.05%)	0.014	1.518214
**Marital status**					
Single	5 (26.32%)	12 (63.16%)	2 (10.53%)		
Married	19 (14.39%)	99 (75.00%)	14 (10.61%)	0.221	0.6248475
Divorce	2 (8.70%)	17 (73.91%)	4 (17.39%)	0.326	0.649636
Separate	0 (0.00%)	3 (60.00%) |	2 (40.00%)	0.786	0.2630051
**Socioeconomic**					
Education level					
Informal	2 (5.71%)	30 (85.71%)	3 (8.57%)		
Illiteracy	17 (17.71%)	68 (70.83%)	11 (11.46%)	0.074	−1.984648
Primary	4 (13.79%)	21 (72.41%)	4 (13.79%)	0.190	−2.066674
Secondary	3 (15.79%)	13 (68.42%)	3 (15.79%)	0.138	−2.348263
Bachelor	0 (0.00%)	1 (50.00%)	1 (50.00%)	0.311	−3.959646
**Gender**					
Male	1 (3.85%)	24 (92.31%)	1 (3.85%)		−1.619828
Female	25 (16.13%)	109 (70.32%)	21 (13.55%)	0.032	
**Household income per month**					
Middle income	6 (6.90%)	72 (82.76%)	9 (10.34%)	0.003	1.066451
High income	0 (0.00)	4 (100.00%)	0 (0.00%)	0.982	13.65555
**Accessibility**					
Accessible	4 (5.80%)	61 (88.41%)	4 (5.80%)		
Un accessible	22 (19.64%)	72 (64.29%)	18 (16.07%)	0.001	−2.266518
**Affordability factor**					
Affordable	3 (6.12%)	45 (91.84%)	1 (2.04%)		
Un affordable	22 (16.79%)	88 (67.18%)	21 (16.03%)	0.003	−2.749899

Abbreviation: IDP, internally displaced person.

#### 3.1.3 Multivariate OLR Analysis

Table 3 presents the results of the multivariate OLR, which was used to identify the factors significantly predicting the utilization of healthcare services. After controlling for all variables in the model, affordability, accessibility, and gender emerged as statistically significant determinants. The most powerful predictor of healthcare utilization was affordability. The finding that services were perceived as “unaffordable” was highly significant (*p* < 0.001) and was associated with a large negative coefficient (−2.270762). This strong negative coefficient means that the odds of an individual utilizing healthcare services are substantially lower if they perceive the services as unaffordable, compared to those who find them affordable. This highlights cost as the most critical barrier in this community. Similarly, accessibility was a significant factor. The coefficient for “un accessible” was −1.441912 (*p* = 0.025), indicating that individuals who reported difficulty in physically reaching healthcare facilities had significantly lower odds of using them compared to those who found them accessible.

**TABLE 3:** Multivariate ordinal logistic regression.

Variable	*p* value	Coef.
**Accessibility factor**		
Accessible		
Un accessible	0.025	−1.441912
**Affordability factor**		
Affordable		
Un affordable	0.000	−2.270762
**Socioeconomic factor**		
Education level		
Illiteracy	0.349	−0.5423792
Primary/Intermediate	0.255	−0.7948083
Secondary	0.102	−1.253905
Bachelor degree	0.406	−1.275629
Gender		
Female	0.030	−1.730924

A crucial and statistically significant finding was related to gender. The negative coefficient for females (−1.730924, *p* = 0.030) indicates that, when all other factors are held constant, women in this sample had significantly lower odds of utilizing healthcare services compared to their male counterparts. This suggests the presence of specific, unaddressed barriers that disproportionately affect women's ability or willingness to seek care. In contrast to these findings, an individual's education level was not found to be a statistically significant predictor of healthcare utilization in this model. Although negative coefficients were observed for all educational categories, their *p* values were well above the 0.05 threshold for significance (*p* = 0.349 for illiteracy, *p* = 0.102 for secondary, etc.). This means we cannot conclude that education has a real effect; the observed differences are likely due to random chance. Therefore, after accounting for the more powerful effects of cost, access, and gender, educational attainment did not independently influence healthcare‐seeking behavior in this population.

## 4 Discussion

A central finding of this study is the alarmingly high proportion of respondents (73.48%) reporting low utilization of healthcare services. Our multivariate regression analysis provides a clear explanation for this trend, moving beyond description to identify the underlying drivers. The model demonstrates that this widespread underutilization is not random but is significantly predicted by the formidable barriers of affordability and accessibility. In essence, the high prevalence of low utilization is a direct reflection of a system where prohibitive costs and physical distance are the primary determinants of who receives care. This linkage underscores that for the vast majority of this IDP population, the decision to seek healthcare is overwhelmingly constrained by these structural and economic obstacles.

The significance of physical accessibility as a barrier is well‐documented in diverse settings. Our findings are consistent with large‐scale research in Indonesia [14] and Nepal [15], where geographic distance was found to be a major impediment to healthcare utilization. The study in Nepal further quantified this, suggesting that improved transportation could dramatically increase population coverage [15]. Similarly, research in Ghana highlighted how access diminishes significantly for higher level care, with only a fraction of the population living within the recommended distance for secondary and tertiary facilities [16]. However, the role of distance is not always paramount; a study in a semi‐urban setting in Nigeria found that it was not a significant barrier, suggesting that other factors like the attitude of health personnel can be more influential [17, 18]. This highlights that although accessibility is a common challenge, its relative importance is context‐dependent.

Likewise, the powerful influence of affordability in our study aligns with global evidence. Our finding that out‐of‐pocket expenses for registration, labs, and medicine were a significant deterrent is mirrored by research in the Western Cape, South Africa, which also identified a strong association between affordability and health service use [19]. This financial barrier is a near‐universal challenge for displaced populations who have lost livelihoods and social safety nets. However, what makes this a unique and acute barrier for IDPs in Somaliland is the protracted nature of displacement combined with a fragile public health system lacking robust social safety nets. Unlike IDP contexts with large‐scale, sustained humanitarian operations providing free services, the camps in Hargeisa exist in a “post‐emergency” phase. Here, IDPs must compete for services in a heavily privatized market with inconsistent support, as noted in recent displacement assessments (NDRA 2023). This forces a direct trade‐off between healthcare and other essential needs, making affordability not just a barrier, but often an insurmountable wall.

Perhaps the most critical and context‐specific finding relates to the significant gender disparity, where men in our sample had significantly lower odds of utilizing healthcare services. This underutilization is likely rooted in cultural norms that position men as stoic providers, discouraging them from seeking care for non‐acute conditions. Complementing this, a critical structural barrier exists for men engaged in informal daily labor, as taking time off for a clinic visit means a direct loss of essential household income. Our finding aligns with research in other African contexts; for example, a study in Kenya also found that being male was associated with lower utilization [20], whereas research in Ethiopia showed that females were significantly more likely to access care, which similarly points to men as the less frequent users [21]. These combined cultural and economic pressures create a powerful disincentive that appears to be the dominant gender‐related barrier in this specific context, underscoring that gender dynamics are highly localized and must be understood within their specific socioeconomic environment. Research in the region reveals that healthcare barriers for internally displaced people are complex and context‐dependent, ranging from primarily financial (62.5%) and physical transport (37.9%) obstacles in **Sudan** to the critical challenge of political fragmentation in **Syria**, where access is dictated by territorial governance [22, 23, 24, 25].

Finally, although our model did not find income to be a statistically significant independent predictor, this does not diminish the role of poverty. Instead, it suggests that within this specific low‐income population, the effects of poverty are better captured by the more immediate barriers of affordability and accessibility. This is consistent with findings from Bangladesh, where income level was not significantly associated with healthcare utilization [20], but contrasts with the strong income effects seen in Ethiopia. This variability across studies reinforces that in the Somaliland IDP context, the practical barriers of direct costs and physical access are the ultimate gatekeepers to healthcare, even more so than underlying income levels.

Our findings demand a coordinated policy and programmatic response from all key actors. The government, through the MoHD, is responsible for the high‐level policy, specifically by formulating a national framework that ensures affordability through subsidized care or a dedicated health fund for IDPs. In parallel, UN bodies and partner NGOs are crucial for on‐the‐ground implementation, designing and deploying targeted interventions like mobile clinics and community health worker programs to overcome physical accessibility barriers. This strategic division of labor policy led by the government and implemented by UN/NGO partners is essential to create a tangible and sustainable improvement in healthcare access for these vulnerable communities.

## 5 Future Research

Building upon the associations identified in this cross‐sectional assessment, future research should prioritize a longitudinal study design to investigate the causal factors driving the underutilization of health services in the Ayah 4 and Nasahablood IDP camps. A longitudinal cohort study, tracking the same households over several months or years, would allow researchers to move beyond correlation and establish the temporal sequence of events. Such a study could effectively measure how changes in specific variables, such as household income, food security status, trust in healthcare providers, or the implementation of new community health initiatives, directly impact health‐seeking behaviors over time. By examining these dynamic relationships, future research can provide definitive, evidence‐based insights into the causal pathways of low service utilization and inform the design of more targeted and effective public health interventions for these vulnerable populations. Although this study identified key statistical predictors of low healthcare utilization, it is recommended that future research employ a mixed‐methods approach to build upon these findings. The integration of qualitative methods, such as in‐depth interviews or community focus groups, is essential for exploring the lived experiences behind the quantitative trends. This would provide a deeper, more nuanced understanding of community perspectives on affordability, accessibility, and gender‐related barriers, capturing the personal narratives and social contexts that a purely statistical analysis cannot. By combining quantitative and qualitative data, future studies can develop a more holistic picture and inform the design of interventions that are not only evidence‐based but also culturally sensitive and responsive to the community's specific needs.

## 6 Conclusion

In conclusion, this study reveals that healthcare utilization among the Nasahablod C and Ayah 4 IDP populations is critically low, not due to a lack of need, but because of formidable structural barriers: affordability, physical accessibility, and gender‐specific constraints. Simply having a health facility nearby is insufficient when the population cannot afford its services, reach it safely, or feel culturally comfortable seeking care. Therefore, to translate these findings into meaningful impact, we recommend a coordinated, multilevel response. Policymakers, particularly the Somaliland Ministry of Health Development, should spearhead this by formulating a national policy that designates IDPs as a priority vulnerable group, mandating the creation of a health fund or a user fee‐exemption scheme to remove the primary barrier of cost. In parallel, NGOs and UN bodies must tackle physical access by designing and implementing targeted interventions, such as deploying mobile health clinics on a regular schedule and establishing a network of trained community health workers based within the camps. Finally, healthcare providers at the facility level must adapt their service delivery to be more gender‐sensitive and culturally competent, specifically by designing outreach campaigns to address the factors hindering men's health‐seeking behavior and offering flexible clinic hours to accommodate the schedules of daily laborers. Implementing these specific, interlinked recommendations is essential to dismantle the obstacles that prevent this vulnerable population from realizing their fundamental right to health.

## 7 Study Limitation

The authors acknowledge several limitations inherent in this study's design. First, the cross‐sectional nature of the study precludes the establishment of causal relationships; findings should therefore be interpreted as strong associations rather than direct cause‐and‐effect. Second, although the findings are robust for the Nasahablood and Ayah 4 camps, their generalizability to the broader IDP population across Somaliland is limited, as displacement contexts and healthcare challenges may differ significantly in other regions. Finally, the reliance on self‐reported data from a structured questionnaire introduces the potential for recall bias, as participants may not accurately remember past events, and social desirability bias, where responses may be influenced by perceived social norms. These limitations underscore the value of the recommended future longitudinal and mixed‐methods research to build upon this foundational study.

## Author Contributions

Ayan Hussein Korse conceptualized the manuscript. After Ayan Hussein Korse and Vitalis Okoth Odero analyzed the data, Hana Mahdi Dahir, Mohamed Said Hassan, and Khadar Abdi Ibrahim verified the findings. Ayan Hussein Korse created the figure. Ayan Hussein Korse and Vitalis Okoth Odero drafted this manuscript. Mohamed Said Hassan, Khadar Abdi Ibrahim, and Hana Mahdi Dahir oversaw the entire study and provided critical revisions to the manuscript for significant intellectual content. All authors have approved the final draft for publication.

## Ethics Statement

No human body samples were used in this investigation, and no personally identifiable information was gathered. Nonetheless, the highest ethical standards were used throughout the study. The Amoud University School of Medicine ethically approved the study with reference 0300‐AU‐REC‐2024.

## Consent

The participants were informed of the study goals, confidentiality, and consent before the survey.

## Conflicts of Interest

The authors declare no conflicts of interest.

## Novelty of the Study

This study represents the first comprehensive investigation into factors influencing healthcare service utilization among internally displaced persons (IDPs) in the Nasahablood and Ayah 4 camps in Hargeisa, Somaliland, offering crucial insights into a population facing unique healthcare challenges. The findings have the potential to inform targeted, culturally appropriate interventions and guide policies aimed at improving healthcare access for IDPs in Somaliland and similar settings, thereby contributing both regionally and globally to the understanding of healthcare utilization in displaced communities.

## Data Availability

The corresponding author can provide the primary data to support the study's findings upon request.
